# School Violence Among a Nationally Representative Sample of Adolescents in Chile

**DOI:** 10.3389/fpubh.2020.00046

**Published:** 2020-02-26

**Authors:** Anne Abio, Jennifer K. Wilburn, Masood Ali Shaikh, Michael Lowery Wilson

**Affiliations:** ^1^Injury Epidemiology and Prevention Research Group, Division of Clinical Neurosciences, Turku Brain Injury Centre, Turku University Hospital and University of Turku, Turku, Finland; ^2^Injury Epidemiology and Prevention Research Unit, Heidelberg Institute of Global Health (HIGH), University of Heidelberg, Heidelberg, Germany

**Keywords:** school violence, adolescent health, bullying, physical fighting, Chile

## Abstract

**Background:** School violence is widely acknowledged as a public health problem with considerable consequences on student learning and social development. There are also a wide range of health consequences. A large share of previous research on school violence has focused on populations in the global north, with significant gaps in the state of knowledge in the world's emerging economies. To this end, the present study provides an examination of correlates for school-based violence in Chile using a nationally representative cohort.

**Methods:** Six independent variables were considered (age, sex, physical activity, sedentary life style, bullying victimization, food insecurity) within a logistic regression model to ascertain the strength and direction of associations with physical fighting.

**Results:** Among the surveyed students, ~13.08% reported being involved in two or more physical fights during the twelve month recall period. Males were significantly over represented among those reporting being involved in a fight OR 2.91 (CI = 1.98–4.27). Those who reported experiencing food insecurity were 5.29 (CI = 1.43–19.50) times more likely to have been involved in a physical fight. Students who reported being bullied were 2.41 (CI = 1.67–3.47) times more likely to have been involved in physical fights. While age provided protection from involvement in physical fights with an adjusted odds ratio of 0.91 (CI = 0.84–0.98).

**Conclusion:** Consistent with previous research, our results suggest that the use of school-based interventions that target multiple risk behaviors may be helpful in reducing rates of physical fighting.

## Introduction

Interpersonal violence is a serious public health problem in the Americas, with youths being the most affected age group. Majority of the efforts deterring interpersonal youth violence target persons aged 10–24 years, although the “youth” have been defined as individuals aged between 10–29 years (1, 2). It is estimated that interpersonal youth violence contributes to 15.8% of all disability-adjusted life years for this age group and 30.7% of total years of life lost (3).

Globally, youth violence was the fourth leading cause of mortality among the 15–19 year old adolescents and second among males in 2013 (3, 4). Violence among adolescents includes but is not limited to physical fights, intimidation or threats, bullying, sexual and emotional abuse, among others (5). Bullying victimization and various forms of stress stimulate mental health problems and disorders among adolescents and youth (6–9). These factors are likely to have a substantial mental, social, and personal impact, which may be manifested through the high prevalence of physical fighting among this age group. Previous studies have found that drug abuse, smoking, alcohol, bullying victimization, and poor negative behavior control are some of the common factors that are associated with physical fighting among adolescents (4, 10–13). Moreover, other seemingly minor types of violence are also strong indicators of youth violence; these include causing injuries to other persons, corporal punishments and other aggressive behavior (2). On the other hand, parental supervision, helpful peers, positive school environments and older age have been found to have a protective effect (4, 11, 13–15). Sedentary behavior and physical activity have had varying results in relation to physical fighting, with some studies finding sedentary behavior to be a risk factor while physical activity was protective in one study and not the other (14, 16). Factors that influence youth violence in the Americas are generally related to many societal pressures that youths experience on a day to day basis. Some of these factors include rapid urban growth, poverty coupled with income inequality and rampant drug trafficking (2). The violence may also be exacerbated by poverty with 11% of the population living on less than 3.2 US$ a day by 2013 Latin America (17). Throughout the region, youth gangs are ubiquitous and well-recognized as a serious problem (2). Puerto Rico, Colombia, El Salvador, the Bahamas and Brazil experience the highest proportion of youth deaths from homicides in the Americas (3). While it is well-known that Latin America has extremely high rates of youth violence overall, few studies have empirically examined the situation in South American contexts.

In Chile, it is thought that youth gangs came into predominance following the economic crisis that characterized the 1980s (18). A study conducted in 2004 in Chile found the prevalence of physical fights among adolescents to be 40.7% (10). The investigation of the social context and influences associated with physical fighting among adolescents are important in highlighting the factors that potentially affect their behaviors in Chile. This study examines the extent to which previously identified correlates in similar settings elsewhere were associated with violent behavior among school-attending adolescents in Chile. Specifically, we examined the effect of age, gender, physical activity, sedentary life style, victimization, and food insecurity as a proxy for socioeconomic status (11, 12, 12–14, 19, 20). The aim of the study was to examine the risk factors associated with physical fighting among school attending adolescents in Chile.

## Methods

### Setting

This study was based on data collected in the Republic of Chile, a South American country bordering the Pacific Ocean on the west and Argentina on the east. It is a predominantly urban country, with a population of ~18,209,068 as of 2016 (17). The country has a Gross National Income per capita of USA $14,640 (2018) and as such is classified as a high-income country (17).

### Data Description

The data for this study were derived from the Chilean contribution to the 2013 Global School-based Health Survey (GSHS). The methodology for the GSHS was developed collaboratively by the World Health Organization and the United States Centers for Disease Control and Prevention. All data used in this study were freely available and accessible via the WHO website (https://www.who.int/ncds/surveillance/gshs/datasets/en/). The survey was administered to school attending adolescents in grades 7 and 8 “basico,” and 1 to 4 “medio” (usually attended by 13–17 year olds). It collected self-reported information on indices pertaining to health risk behaviors. The 2013 Chile GSHS employed a two-stage cluster sampling design to generate data representative of all students in each of the aforementioned grades in Chile. At the first stage, schools were selected with probability proportional to their enrollment size. In the second stage, classes within these schools were randomly selected with all students in the selected classes being eligible to participate. This process resulted in 2,049 students aged 11 years or younger to 18 years or older (49.93% female) having completed the questionnaire. The overall response rate was 60%, with 90% of schools and 67% of students responding to the survey. The analysis presented in this paper excluded one case that had missing information on physical fighting. However, other missing information included 16 records missing gender and 1 record missing age (0 records missing both) were included in the analysis.

### Measurements

The dependent variable “physical fighting” was derived from the survey question:

“*During the past 12 months, how many times were you in a physical fight?*” Response

options ranged from “*0 times”, “1 time”, “2 or 3 times”, “4 or 5 times”, “6 or 7 times”, “8 or 9 times”, “10 or 11 times*” or “*12 or more times*”. For the purpose of our analyses, participants were classified as having participated in a physical fight if they reported being in two or more fights (*N* = 280) in order to identify problematic fighting behavior. If none or one fight was reported, participants were classified as not participating in a physical fight (*N* = 1,768).

We investigated six independent variables to screen for statistically significant associations with the dependent variable; five at the individual level (age, sex, physical activity, sedentary life style, and bullying victimization) and one independent variable at the social level (food insecurity). All independent variables, apart from age, were dichotomized according to the distribution of the data in order to facilitate analysis (Table 1).

**Table 1:** Independent variable derivation from GSHS survey data Chile 2013.

**Survey question**	**Coding**	**Variable**
**Individual-level variables**		
How old are you?	12–18 years (coded continuous)	Age
What is your sex?	Male (1)Female (0)	Sex
During the past 30 days, on how many days were you bullied?	0 days (0) 1 or more days (1)	Bullying victimization
During the past 7 days, on how many days were you physically active for a total of at least 60 min per day?	3 days or less (0) 4 days or more (1)	Physical activity
How much time do you spend during a typical or usual day sitting and watching television, playing computer games, talking with friends, or doing other sitting activities such as surf the internet?	2 h or less (0) 3 h or more (1)	Sedentary lifestyle
**Social-level variable**		
During the past 30 days, how often did you go hungry because there was not enough food in your home?	Most of the time/always (1) Never/rarely/sometimes (0)	Food insecurity

#### Food Insecurity

Associations with having gone hungry within the 30 days preceding the survey were examined using the question: “During the past 30 days, how often did you go hungry because there was not enough food in your home”? The responses were: “Never”; “rarely”; “sometimes”; “most of the time”; or “always.” These were dichotomized into “yes” corresponding to “most of the time/always” and “no” corresponding to “never/rarely/sometimes.”

#### Bullied

Being bullied was examined using the question: “During the past 30 days, on how many days were you bullied?” The responses were: “0 days”; “1 or 2 days”; “3 to 5 days”; “6 to 9 days”; “10 to 19 days”; “20 to 29 days”; “All 30 days”. These were dichotomized into “0 days” and “1or more days.”

#### Physical Activity

Physical activity was examined using the question: “During the past 7 days, on how many days were you physically active for a total of at least 60 min per day?” The responses were: “0 days”; “1 day”; “2 days”; “3 days”; “4 days”; “5 days”; “6 days”; “7 days.” These were dichotomized into “3 days or less” and “4 days or more.”

#### Sedentary Lifestyle

Sedentary lifestyle was examined using the question: “How much time do you spend during a typical or usual day sitting and watching television, playing computer games, talking with friends, or doing other sitting activities such as surf the internet?” The responses were: “Less than 1 h per day”; “1 to 2 h per day”; “3 to 4 h per day”; “5 to 6 h per day”; “7 to 8 h per day”; “More than 8 h per day.” These were dichotomized into “2 h or less” and “3 h or more.”

### Statistical Analysis

Since there were only four students who were either 11 years old or younger, they were combined with the next age category of 12 years old for analysis. Univariate analyses entailed calculating cumulative percentages of all variables studied. Next, status in physical fights involvement (involvement in one or no fights vs. or more fights) was compared with all the independent variables. Categorical and continuous variables were explored taking the survey design into account, using the survey versions of a *t*-test and chi-square test. Independent variables found to be statistically significant at the *p*-value of <0.05 in the bivariate analysis were included in the multivariable analysis using multiple logistic regression. Odds Ratios (OR), including their 95 and 99% confidence intervals, are reported for the association with physical fight involvement. Stata version 15 (StataCorp LP, 2017) was used for the data analysis.

## Results

Within the recall period, 13.08% (unweighted count: 280) of participants reported being involved in two or more physical fights. The mean age of the sample was 15.1 years old (SD: 1.8) and the majority (72.1%) were male. Bullying victimization was reported by 13.0% of respondents. Only 35.7% reported being physically active for at least 60 min per day in the past seven days, for 4 or more days. Sedentary life style was defined as spending three or more hours on a usual day sitting and watching television, playing computer games or doing other sitting activities, and was reported by 53.7% of respondents. Moreover, 1.8% reported that during the past 30 days, they either went hungry all or most of the time because there was not enough food in their home.

### Bivariate Analysis

The bivariate analyses show that within all the selected variables, significant differences existed between participants who had been involved in physical fights and those who had not (Table 2). There was significant difference in the ages of students who were involved in physical fighting when compared with those who were not (14.89 vs. 15.16 respectively, *P*-value: 0.025). In the bivariate analyses, the majority of adolescents reporting 2 or more fights were males (72.1%). Sex (male) and bullying victimization were the most significant variables (*P*-values: <0.001) associated with physical fighting.

**Table 2:** Distribution of selected factors according to categories of physical fighting among school-attending adolescents in Chile, GSHS 2013.

**Variable**	**Not involved in physical fights (*n* = 1,768)**	**Involved in physical fights (*n* = 280)**	***P*-value**
Age (SD)	15.16 (1.79)	14.89 (1.79)	0.025
Sex (male)	46.3	72.1	<0.001
Food deprivation	1.3	5.3	0.016
Bullying victimization	11.1	26.1	<0.001
Physical Activity	34.3	45.5	0.029
Sedentary	52.5	61.6	0.014

*All variables are expressed as proportions (in %) with the exception of age, which is expressed as mean and standard deviation*.

### Multivariable Analyses

An age and sex adjusted analysis (Table 3) was conducted for all variables used. The analyses revealed statistically significant associations for all selected variables, with the exception of physical activity (*P*-value: 0.232). Using 95% confidence intervals, sex (male) and bullying victimization were found to be the most significant variables. Males were at least three times more likely to have been involved in physical fights in the past 12 months compared to females (OR 3.03, CI = 2.10–4.34). Adolescents who experienced bullying were twice as likely to have been involved in physical fights than those who had never experienced bullying (OR 2.59, CI = 1.83–3.67). Adolescents who went hungry all or most of the time were five times more likely to have been involved in physical fights than those who did not experience food deprivation (OR 5.15, CI = 1.32–20.16). However, using 99% confidence intervals, i.e. using a significance level of <0.01, food deprivation and age were not found to be statistically significantly associated with physical fighting (99% CI = 0.81–32.82, *p* = 0.021), and (99% CI = 0.83–1.00, *p* = 0.012), respectively.

**Table 3:** Multivariable analysis of physical fighting among school-attending adolescents in Chile, GSHS 2013.

**Variable**	**OR**	**95% CI (99% CI)**	***p*-value**
Sex	3.02	2.10–4.34 (1.85–4.94)	<0.001
Age	0.91	0.85–0.98 (0.83–1.00)	0.012
Food deprivation	5.15	1.32–20.16 (0.81–32.82)	0.021
Bullying victimization	2.59	1.83–3.67 (1.61–4.16)	<0.001
Sedentary	1.59	1.18–2.13 (1.06–2.37)	0.004
Physical Activity	1.28	0.85–1.93 (0.73–2.24)	0.232

*OR, Odds Ratio; 95 and 99% CI, 95 and 99% Confidence Intervals*.

*All estimates are adjusted for age and sex, while age and sex are adjusted for each other*.

A final model adjusted for all associated covariates used (Table 4). When compared to those who did not report being involved in a physical fight in the past 12 months, those who had been involved were more likely to be males (OR 2.91; CI = 1.98–4.27), be food deprived (OR 5.29; CI = 1.43–19.50), have been bullied (OR 2.41; CI = 1.67–3.47), and lived a sedentary lifestyle (OR 1.53; CI = 1.09–2.16). Younger age provided protection from involvement in physical fights (OR 0.91; CI = 0.84–0.98). There was no statistically significant association between physical activity and involvement in physical fighting (OR 1.42; CI = 0.91–2.22). The goodness-of-fit test revealed that this multivariable logistic model was a good fit for physical fighting in Chilean school students (F: 0.15, *p*-value: 0.99). Using the *p*-value of <0.01 i.e., 99% confidence intervals, only sex and bullying victimization were found to be statistically significant.

**Table 4:** Outcomes of multivariable analysis of variables associated with physical fighting attempts among school-attending adolescents in Chile, GSHS 2013.

**Variable**	**Adjusted odds ratio**	**95% CI (99% CI)**	***P*-value**
Sex	2.91	1.98–4.27 (1.72–4.90)	<0.001
Age	0.91	0.84–0.98 (0.82–1.00)	0.013
Food deprivation	5.29	1.43–19.50 (0.90–31.07)	0.015
Bullying victimization	2.41	1.67–3.47 (1.46–3.95)	<0.001
Physical Activity	1.42	0.91–2.22 (0.78–2.60)	0.116
Sedentary	1.53	1.09–2.16 (0.96–2.43)	0.017

*Only those factors found statistically significant in bivariate analysis were used in this model*.

*CI, Confidence Interval*.

*All estimates are adjusted for all variables listed in the table*.

## Discussion

Within the study and recall period, we found that ~13.1% of school-going adolescents had been involved in physical fights in the 12 months prior to being surveyed. This is significantly lower when compared to a previous Chile GSHS study conducted in 2004 which reported a prevalence of 40.7% (10). This is interesting as research has shown that in general, violence in Chile has shown a clear upwards trend since the end of the 1990s and the early 2000s (21–24). However, during this time, two national crime-fighting plans were implemented and one, aimed specifically at reducing delinquency rates, was found to be very successful (23).

The rate was also lower than reported rates among Portuguese (19) and Israeli adolescents (20) and significantly lower when compared to rates in some low-middle income countries, such as Egypt (14) and Ghana (11), and upper-middle income countries, such as Malaysia (1). Interestingly, it is also lower than some rates in other countries in the Americas. Argentina and Uruguay are high income countries (like Chile) with reported rates of physical fighting of 39.1% and 25.9% in 2012, respectively (25, 26). It is difficult to make a direct comparison, however, as most of the previous studies considered students who had been involved in a physical fight at least once or more times during the 12 months prior to being surveyed, as experiencing physical fighting. This contrasts with the current study which classified students who had been in a fight only one time explicitly as not problematic physical fighting.

The Chilean prevalence is similar to studies conducted in other high income countries, such as the US and Thailand at 14% (3, 14). The estimate from this study is not very unexpected when deduced through a socioeconomic and cultural context. According to some studies, poverty may be a factor that contributes to societies tolerating violence (27, 28). As Chile is a high-income country, one would have expected a similar prevalence as those of other high income countries. Interestingly, income inequality in Chile is quite high (47.7 in 2015), but consistent with other high-income countries in South America (ex. Argentina−42.4 and Uruguay−39.7) (29). This tends to occur in areas where there is significant social and economic inequality. However, similar to previous studies, it was not possible to examine whether income levels within the region affected the current data, as the Global School Health Survey did not collect data on income (14).

The differences in the prevalence of physical fighting between Chile, South American countries and some high-income countries highlights the potential societal contrasts in the tolerance of violence among them. Globally, Chile has a low rank on the crime scale. It had an annual murder rate of 4.6 per 100,000 in 2015 (30) and is considered to be relatively safe, with comparatively less violent crime than in other Latin American countries (31).

We found that, consistent with the literature, there was a statistically significant relationship between gender and physical fighting behavior. In the current study, 72% of those who had been involved in fights were male, a higher estimate than in the previous study at 54% (32). According to previous studies, males have been found to get more involved in aggressive behavior and physical fighting compared to females in various settings (4, 10, 12–14, 16, 33). In Chile, gender-based violence against women is common and the culture around gender is very patriarchal (34, 35). A 2004 study determined that 50% of married women had experienced domestic violence particularly, physical and emotional abuse (34). The important factors for violence against women are mainly economic, with cultural norms that require female obedience and submission (35). Girls are typically expected to exhibit behaviors ascribed with being feminine to avoid aggressive behaviors, though recent studies have suggested the trend is changing (35).

Consistent with previous research, having been bullied was associated with physical fighting (10–14, 36, 37). While it was not possible to determine if students were engaging in physical fighting as a defense mechanism against bullies, these results support the evidence that adolescent involvement in physical fights is associated with other types of negative peer behavior.

Hunger was found to be significantly associated with physical fights. This is consistent with previous studies (16, 33). However, no significant association was found when the confidence interval was increased to 99%. There is relatively limited research analyzing the impact of hunger on physical fighting (15). Chile went through a rapid progression of nutritional transition and malnutrition was nearly eradicated by the end of the 1980s (38). The lack of significance found in this study may, in part, be due to the fact that the current study took place in a high-income country context and hunger is usually closely tied with poverty, which more often occurs in low and middle income countries (15).

Significant differences were also noted among those who reported low physical activity and a sedentary lifestyle, consistent with previous studies (14, 16, 39, 40). However, no significant association was found for either after increasing the confidence interval to 99%. These findings contribute to a small body of literature on these factors and how they influence interpersonal violence among adolescents (14, 41–43). Compared with other American countries, the risk factors associated with non-communicable diseases are higher in Chile compared to the regional average (38). As Chile's diet became progressively westernized in the early 1990s, physical activity levels decreased overall due to urbanization (38). At the same time, it is possible that the overall low rates of physical activity are the byproduct of an unsafe environment. Neighborhood violent crime has been seen as a barrier to actively engaging in outdoor physical activity in some settings (44).

The results from this study provide evidence demonstrating that school environments may represent strategic focal points for initiatives aimed at reducing bullying victimization and perpetration. There exists some support in existing literature, for the reduction of interpersonal aggression within school environments that encourage greater participation in physical activity. Physical activity interventions are part of overall health and well-being promotion in schools and have been shown to also have positive effects on student mental health.

## Strengths and Limitations

This study used data from the Chile contribution to the 2013 Global School-based Health Survey (GSHS) and was representative of all school-attending adolescents aged 11–18 in Chile. The sample size was large enough to allow for statistical analyses to generate valid results. A multi-stage sampling process was used and thus reduces sampling bias. The survey questionnaire was piloted in cross-national and -cultural settings and, all surveys were carried out in a controlled school-based environment which ensured anonymous responses to the questionnaires. However, the survey does not reach adolescents who were potentially not present on the day of the survey (due to illness or otherwise) or the small minority of adolescents that do not attend school. School attendance in Chile in general is close to 100% (45). Thus, we anticipate the effect of school absence on the results, where present, to be minimal. As the data are cross-sectional, it is difficult, if not impossible, to determine the direction of influence between variables or to determine causal relationships.

Another potential limitation with using the GSHS data is that it is skewed toward only capturing physical violence as opposed to relational violence, even though both are considerably harmful. Relational violence mainly but not exclusively involves covertly or directly manipulating someone via their relationships with the aim of damaging their social status or reputation (such as name calling, teasing and having rumors spread); this is typically higher among females and can have long-term negative psychological effects (46).

Even though this was an anonymous survey, the answers to the questionnaires were self-reported and so maybe subject to cultural pressures and non-response bias. Although the design intended to be administered cross-culturally, some questions may have been misinterpreted and/or misunderstood by the respondents.

## Conclusions

There is limited information about the epidemiology of physical fighting among youths in Chile. Physical fighting was found to be associated with a number of factors within the targeted study population in Chile. Consistent with previous research, our results suggest that the use of school-based interventions that target multiple risk behaviors could be beneficial to reducing physical fighting among adolescents.

## Data Availability Statement

Publicly available datasets were analyzed in this study. This data can be found here: https://extranet.who.int/ncdsmicrodata/index.php/catalog/421/get_microdata.

## Ethics Statement

The studies involving human participants were reviewed and approved by Ministerio de Salud de Chile. Written informed consent to participate in this study was provided by the participants' legal guardian/next of kin.

## Author Contributions

Conceptualization and the original draft preparation was done by MS and AA. Formal data analysis and methodology was done by MS. Validation and review and editing of the manuscript was done by MW and JW. Supervision was done by MW.

### Conflict of Interest

The authors declare that the research was conducted in the absence of any commercial or financial relationships that could be construed as a potential conflict of interest.

## References

[1] CDC Preventing Youth Violence. Available online at: https://www.cdc.gov/violenceprevention/youthviolence/definitions.html (accessed June 12, 2018).

[2] Atienzo EE Baxter SK Kaltenthaler E. Interventions to prevent youth violence in Latin America: a systematic review. Int J Public Health. (2017) 62:15–29. 10.1007/s00038-016-0909-6 27766375 PMC5288433

[3] Mokdad AH Forouzanfar MH Daoud F Mokdad AA El Bcheraoui C Moradi-Lakeh M. Global burden of diseases, injuries, and risk factors for young people's health during 1990-2013: a systematic analysis for the Global Burden of Disease Study 2013. Lancet. (2016) 387:2383–401. 10.1016/S0140-6736(16)00648-6 27174305

[4] Yang L Zhang Y Xi B Bovet P. Physical fighting and associated factors among adolescents aged 13-15 years in six Western Pacific Countries. Int J Environ Res Public Health. (2017) 14:1427. 10.3390/ijerph14111427 29160819 PMC5708066

[5] Weaver K Maddaleno M. Youth violence in Latin America: current situation and violence prevention strategies. Rev Panam Salud Publica. (1999) 5:338–43. 10.1590/S1020-49891999000400025 10355334

[6] Acquah EO Topalli P-Z Wilson ML Junttila N Niemi PM Adolescent loneliness and social anxiety as predictors of bullying victimization. Int J Adolesc Youth. (2015) 21:320–31. 10.1080/02673843.2015.1083449

[7] Wilson ML Celedonia KL Kamala BA Patterns, characteristics, and correlates of adolescent bully-victims in urban Tanzania. Soc. Sci. (2013) 2:234–46. 10.3390/socsci2040234

[8] Wilson ML Dunlavy AC Berchtold A Determinants for bullying victimization among 11-16-year-olds in 15 low- and middle-income countries: a multi-level study. Soc Sci. (2013) 2:208–20. 10.3390/socsci2040208

[9] Wilson ML Viswanathan B Rousson V Bovet P. Weight status, body image and bullying among adolescents in the Seychelles. Int J Environ Res Public Health. (2013) 10:1763–74. 10.3390/ijerph10051763 23644826 PMC3709347

[10] Rudatsikira E Muula AS Siziya S. Prevalence and correlates of physical fighting among school-going adolescents in Santiago, Chile. Braz J Psychiatry. (2008) 30:197–202. 10.1590/S1516-44462008000300004 18833418

[11] Acquah E Lloyd J Davis L Wilson M Adolescent physical fighting in Ghana, their demographic and social characteristics. Soc Sci. (2014) 3:227–41. 10.3390/socsci3020227

[12] Mat Hussin SF Abd Aziz NS Hasim H Sahril N. Prevalence and factors associated with physical fighting among Malaysian adolescents. Asia Pac J Public Health. (2014) 26(Suppl. 5):108S−15S. 10.1177/1010539514542423 25038192

[13] Rudatsikira E Siziya S Kazembe LN Muula AS. Prevalence and associated factors of physical fighting among school-going adolescents in Namibia. Ann Gen Psychiatry. (2007) 6:18. 10.1186/1744-859X-6-18 17650328 PMC1947983

[14] Celedonia KL Wilson ML El Gammal HA Hagras AM. Physical fighting among Egyptian adolescents: social and demographic correlates among a nationally representative sample. PeerJ. (2013) 1:e125. 10.7717/peerj.125 24024080 PMC3746957

[15] Acquah EO Wilson ML Doku DT Patterns and correlates for bullying among young adolescents in Ghana. Soc Sci. (2014) 3:827–40. 10.3390/socsci3040827

[16] Jackson DB Vaughn MG. Diet quality and physical fighting among youth: a cross-national study. J Interpers Viol. (2018) 10.1177/0886260518754874. [Epub ahead of print]. 29361879

[17] World Bank Work Bank Open Data. Available online at: https://data.worldbank.org/ (accessed January 24, 2020).

[18] World Bank Youth Gangs and violence in Latin America and the Caribbean: A literature survey. Latin America and Caribbean Region Sustainable Development Working Paper No. 4. (1999). Available online at: http://documents.worldbank.org/curated/en/474291468770479198/pdf/multi-page.pdf (accessed February 02, 2019).

[19] Fraga S Ramos E Dias S Barros H. Physical fighting among school-going Portuguese adolescents: social and behavioural correlates. Prev Med. (2011) 52:401–4. 10.1016/j.ypmed.2011.02.015 21371501

[20] Gofin R Palti H Mandel M. Fighting among Jerusalem adolescents: personal and school-related factors. J Adolesc Health. (2000) 27:218–23. 10.1016/S1054-139X(00)00095-1 10960221

[21] Wilson Center The Prevention of Youth Violence in Latin America: Lessons Learned and Future Challenges. Available online at: https://www.wilsoncenter.org/event/the-prevention-youth-violence-latin-america-lessons-learned-and-future-challenges (accessed February 02, 2019).

[22] UNICEF Chile Annual Report 2017. Available online at: https://www.unicef.org/about/annualreport/files/Chile_2017_COAR.pdf (accessed February 02, 2019).

[23] Overseas Development Institute Policy Brief 8 - Youth Training in Chile and Argentina. Available online at: https://www.odi.org/sites/odi.org.uk/files/odi-assets/publications-opinion-files/4068.pdf (accessed February 02, 2019).

[24] Vergara R Crime prevention programs: evidence from Chile. Dev Econ. (2012) 50:1–24. 10.1111/j.1746-1049.2011.00152.x

[25] WHO Argentina (Buenos Aires) 2012 Fact Sheet. Available online at: https://www.who.int/ncds/surveillance/gshs/Argentina_GSHS_FS_2012_Buenos_Aires.pdf (accessed January 24, 2020).

[26] WHO Uruguay 2012 Fact Sheet. Available online at: https://www.who.int/ncds/surveillance/gshs/2012_Uruguay_GSHS_FS.pdf (accessed January 24, 2020).

[27] Fajnzlber P Lederman D Loayza N Inequality and violent crime. J Law Econ. (2002) 45:1–26. 10.1086/338347

[28] Wilkinson RG Pickett KE. The problems of relative deprivation: why some societies do better than others. Soc Sci Med. (2007) 65:1965–78. 10.1016/j.socscimed.2007.05.041 17618718

[29] World Bank GINI Index. Available online at: https://data.worldbank.org/indicator/SI.POV.GINI (accessed February 04, 2019).

[30] Nation Master Country vs Country: Chile and United States Compared: Crime Stats. Available online at: https://www.nationmaster.com/country-info/compare/Chile/United-States/Crime (accessed February 02, 2019).

[31] OSAC Chile 2018 Crime & Safety Report. Available online at: https://www.osac.gov/Pages/ContentReportDetails.aspx?cid=23485 (accessed February 02, 2019).

[32] PAHO Violence Against Women. Available online at: https://www.paho.org/salud-en-las-americas-2017/?tag=youth-violence (accessed February 02, 2019).

[33] Pickett W Molcho M Elgar FJ Brooks F de Looze M Rathmann K. Trends and socioeconomic correlates of adolescent physical fighting in 30 countries. Pediatrics. (2013) 131:e18–26. 10.1542/peds.2012-1614 23209107

[34] Dianova Violence Against Women in Chile. Available online at: https://www.dianova.ngo/news/violence-against-women-in-chile/ (accessed February 02, 2019).

[35] Delgado JB Meruane PS Varas PR Opazo PM Gender relations and masculinity in northern Chile mining areas: ethnography in schoperías. Etnografica. (2011) 15:413–40. 10.4000/etnografica.1013

[36] Rudatsikira E Muula AS Siziya S. Variables associated with physical fighting among US high-school students. Clin Pract Epidemiol Ment Health. (2008) 4:16. 10.1186/1745-0179-4-16 18510746 PMC2423367

[37] Swahn MH Simon TR Hammig BJ Guerrero JL. Alcohol-consumption behaviors and risk for physical fighting and injuries among adolescent drinkers. Addict Behav. (2004) 29:959–63. 10.1016/j.addbeh.2004.02.043 15219342

[38] Celis-Morales C Salas C Alduhishy A Sanzana R Martínez MA Leiva A. Socio-demographic patterns of physical activity and sedentary behaviour in Chile: results from the National Health Survey 2009-2010. J Pub Health. (2015) 38:e98–105. 10.1093/pubmed/fdv079 26112281

[39] Demissie Z Lowry R Eaton D Hertz M. Associations of school violence with physical activity among U.S. high school students. J Phys Act Health. (2014) 11:705–11. 10.1123/jpah.2012-0191 25078515 PMC10947244

[40] Farhat T Iannotti R Simons-Morton B. Overweight, obesity, youth, and health-risk behaviors. Am J Prev Med. (2010) 38:258–67. 10.1016/j.amepre.2009.10.038 20171527 PMC2826832

[41] Iannotti RJ Janssen I Haug E Kololo H Annaheim B Borraccino A. Interrelationships of adolescent physical activity, screen-based sedentary behaviour, and social and psychological health. Int J Pub Health. (2009) 54:191–8. 10.1007/s00038-009-5410-z 19639256 PMC2732761

[42] Morris RG Johnson MC Sedentary activities, peer behavior, and delinquency among American youth. Crime Delinq. (2010) 60:1–30. 10.1177/0011128710386205

[43] Nelson MC Gordon-Larsen P. Physical activity and sedentary behavior patterns are associated with selected adolescent health risk behaviors. Pediatrics. (2006) 117:1281–90. 10.1542/peds.2005-1692 16585325

[44] Gomez J Johnson B Selva M Sallis J. Violent crime and outdoor physical activity among inner-city youth. Prev Med. (2004) 39:876–81. 10.1016/j.ypmed.2004.03.019 15475019

[45] World Bank School Enrolment, Primary (% gross) - Chile. Available online at: https://data.worldbank.org/indicator/SE.PRM.ENRR?locations=CL (accessed January, 24 2020).

[46] Baldry AC Bullying among Italian middle school students. Sch Psychol Int. (1998) 19:361–74. 10.1177/0143034398194007

